# Structural Elucidation and Storage Stability of Novel Dietary Sulfur Compounds from Radish

**DOI:** 10.3390/foods14183254

**Published:** 2025-09-19

**Authors:** Gyeong A. Jeong, Inae Lee, Jeehye Sung, Chang Joo Lee

**Affiliations:** 1 Department of Food Science and Biotechnology, Wonkwang University, Iksan 54538, Jeonbuk, Republic of Korea; jka0719@naver.com (G.A.J.); ilee3873@khu.ac.kr (I.L.); 2 Department of Food Science and Biotechnology, Kyung Hee University, Yongin-si 17104, Gyeonggi-do, Republic of Korea; 3 Department of Food Science and Biotechnology, Gyeongkuk National University, Andong 36729, Gyeongbuk, Republic of Korea; jeehye@gknu.ac.kr

**Keywords:** dietary sulfur compound, isothiocyanates, freeze-dried radish, structure identification, storage stability, sulforaphane

## Abstract

Radishes (*Raphanus sativus* L.) have attracted interest as functional foods containing various bioactive components. Among them, sulforaphane (SFN), an isothiocyanate compound, is known for its potent antioxidant and anticancer properties. This study aimed to extract, purify, and identify SFN and SFN-derived compound X (SFN-DX), a dietary sulfur-containing bioactive compound, from freeze-dried radishes. High-performance liquid chromatography analysis revealed that the freeze-dried radish extract contained 13.262 mg/g SFN-DX. A major peak was detected with a f of 12.713 min, closely matching that of the SFN standard (RT = 12.883 min). SFN-DX was subsequently isolated with 99% purity through preparative liquid chromatography. Structural elucidation confirmed that SFN-DX is a derivative of SFN and shares characteristic features with isothiocyanates. The storage stability of purified SFN-DX was evaluated under various temperatures (−18 °C, 4 °C, 20 °C, and 40 °C) and storage durations (1 week and 1 month). A significant reduction in compound purity was observed at elevated temperatures and during prolonged storage. Accordingly, −18 °C was identified as the optimal storage temperature for preserving the stability of SFN-DX. Collectively, these findings provide a scientific basis for the efficient extraction, structural analysis, and storage of dietary sulfur-containing compounds derived from radishes.

## 1. Introduction

Over time, the role of food has evolved beyond its primary function of providing essential nutrients for sustaining life to secondary roles influencing sensory qualities such as palatability (e.g., taste, aroma, color, and texture) and ultimately to tertiary functions involving promoting health and preventing disease [1,2]. Recently, these physiological benefits have emerged as key criteria for food selection, driving increased interest in the bioactive compounds present in foods. Furthermore, growing scientific evidence supports the health-promoting effects of various nutrients and non-nutritional phytochemicals in fruits and vegetables, prompting extensive research on these compounds [3].

Cruciferous vegetables (e.g., broccoli, radish, red cabbage, cauliflower, and bok choy, among others) have attracted attention as rich sources of bioactive nutrients and food-derived phytochemicals [4,5]. These vegetables are particularly abundant in antioxidant compounds, including phenolic acids and flavonoids, and contain sulfur-containing compounds, such as glucosinolates and S-methylcysteine sulfoxide, which contribute to their distinctive bitterness and pungency, while also exhibiting diverse biological activities [6,7]. Among them, glucosinolates are the most comprehensively researched for their potential in cancer prevention [7,8]. Upon hydrolysis by myrosinase, glucosinolates are converted into biologically active metabolites, such as isothiocyanates (e.g., sulforaphane) and indoles (e.g., indole-3-carbinol) [3,9]. These metabolites exert protective effects through multiple mechanisms, including cellular defense, carcinogen detoxification, and cell cycle regulation.

Radish (*Raphanus sativus* L.) is a root vegetable belonging to the cruciferous family that is widely cultivated because of its adaptability and versatile applications. Its various cultivars, distinguished by root size, shape, and color, have attracted increasing attention for their functional attributes and industrial applications [7,10,11]. In addition, radish is rich in water, minerals, and vitamins, particularly vitamin C (0.14–0.33 g/kg), which is present in considerable amounts and has been reported to exert beneficial nutritional effects [12]. Beyond its nutritional composition, radish is also recognized for its nutraceutical value, as it contains bioactive compounds such as glucosinolates, isothiocyanates, and polyphenols [13,14]. Radish is abundant in 4-methylthio-3-butenyl isothiocyanate (4-MTBG), an isothiocyanate compound responsible for its pungent flavor that has been linked to a broad spectrum of biological activities, including antioxidant, antibacterial, anticancer, anti-inflammatory, antifungal, and antiviral (e.g., against influenza virus) effects [15,16]. Isothiocyanates (R–N=C=S) are chemically reactive compounds; key dietary isothiocyanates include benzyl isothiocyanate, allyl isothiocyanate, phenyl isothiocyanate, and sulforaphane (SFN) [6,17]. These compounds are classified as dietary organosulfur compounds, with SFN in particular receiving considerable attraction for its potent chemopreventive and anticancer activities [9,18]. In addition, a wide range of in vitro and in vivo studies have demonstrated the broad pharmacological spectrum of SFN [19,20]. In an in vitro Alzheimer’s disease model, SFN reduced Nrf2 promoter DNA demethylation and β-amyloid levels, contributing to anti-inflammatory effects [21]. In an in vivo ovarian cancer model, intraperitoneal administration of SFN (10 mg/kg) overcame cisplatin resistance by enhancing drug sensitivity, thereby exerting a significant anticancer effect [22].

Despite the well-documented bioactivities of sulforaphane and related organosulfur compounds, further investigation into their stability and novel derivatives remains essential. Previous research has suggested that freeze-drying is the most effective method for stabilizing SFN and preserving its maximal content in radishes [23]. In this study, a novel dietary sulfur compound, sulforaphane-derived compound X (SFN-DX), was extracted and isolated from freeze-dried radishes and structurally characterized. In addition, the stability of SFN-DX under various storage temperatures was evaluated to assess its potential application as a functional food ingredient or bioactive compound.

## 2. Materials and Methods

### 2.1. Materials

This study used commercially available Undu-ryeong radish harvested in Gangwon-do in 2022 (Gangwon-do, Republic of Korea) obtained from a local market in its fresh state. Dichloromethane used for extracting dietary sulfur compounds was purchased from Samchun (Gyeonggi-do, Republic of Korea), and the standard reagent DL-sulforaphane (S4441, ≥90% (HPLC)) was purchased from Sigma-Aldrich (St. Louis, MO, USA). All the reagents were of analytical grade.

### 2.2. Sample Preparation and Extraction

Dietary sulfur compounds were extracted from freeze-dried radishes according to the protocol reported by Jeong and Lee [23], a modified protocol based on the method reported by Lee and Kyung [24]. The radishes were washed to remove impurities, cut into 2 × 2 cm^2^ pieces, and stored in a freezer for 24 h. Subsequently, they were freeze-dried using a freeze dryer (LP10, IlShinBioBase Co., Ltd., Dongducheon-si, Gyeonggi-do, Republic of Korea) until their moisture content was below 10%. The dried samples were ground into a powder using a blender. For extraction, 10 g of the powdered sample was mixed with 250 mL of distilled water (DW) and 80 mL of dichloromethane and stirred for 2 h. The mixture was centrifuged at 5000× *g* for 15 min at 4 °C using a centrifuge (Supra R22, Hanil Scientific Inc., Gimpo, Republic of Korea) to separate the organic (dichloromethane) phase. The organic phase was stored in a −70 °C deep freezer (DF8517, IlshinBioBase Co., Ltd., Seoul, Republic of Korea) for at least 2 h, followed by vacuum concentration at 0 °C using a centrifugal evaporator (CVE-3100, EYELA, Tokyo, Japan). The vacuum-concentrated samples were stored at −70 °C to prevent chemical degradation and used for subsequent analyses.

### 2.3. Analytical Methods

#### 2.3.1. High-Performance Liquid Chromatography (HPLC) Analysis

Dietary sulfur compounds extracted from the radishes were analyzed using HPLC with ultraviolet detection (HPLC-UV; Jasco LC-4000, Tokyo, Japan). SFN was selected as the reference compound, while DL-sulforaphane was used as the standard. The analytical method was adapted from Nakagawa et al. [25], with modifications to detect SFN-DX in radish extracts. The analysis sample consisted of a mixture of 10 mg of vacuum-concentrated extract and 1 mL of HPLC-grade acetonitrile (ACN), and the mixture was filtered through a 0.45 μm nylon filter. A reversed-phase C18 column (250 mm × 4.6 mm, 5 μm; YMC-Pack ODS-AQ) was used for separation, and the mobile phase was controlled by combining gradient and isocratic methods. The initial mobile phase was DW:ACN = 81:19 (*v*/*v*), and then isocratic conditions were applied with DW:ACN = 40:60 (*v*/*v*). The flow rate was set at 1 mL/min, the injection volume was 10 μL, and UV detection was performed at 230 nm. The detailed analytical conditions are provided in Table S1 of the Supplementary Materials.

#### 2.3.2. Standard Curve and Quantification for SFN

A standard calibration curve was established to quantify SFN-DX. A standard solution was prepared by dissolving DL-sulforaphane (5 μL/mL in acetonitrile), followed by serial dilution to obtain final concentrations of 0, 25, 50, 75, and 100 ng/mL. The curve was constructed based on the conditions listed in Table S1. Linear regression was applied, and the calibration curve was constructed based on the mean of three replicates for each concentration. All data are presented as mean ± standard deviation (SD). HPLC-grade acetonitrile was used for all the dilutions.

### 2.4. Separation and Purification

#### 2.4.1. Isocratic and Gradient Elution (Recycling Preparative HPLC)

The SFN-DX compound identified by HPLC analysis was isolated and collected using preparative liquid chromatography (Prep-LC; LC-Forte/R-II, YMC, Kyoto, Japan). The separation was performed on a C18 reversed-phase column (JAIGEL-ODS-BP-L, SP-120-15, Japan Analytical Industry Co., Ltd., Tokyo, Japan). The mobile phase started with a composition of DW:ACN = 81:19 (*v*/*v*), which was gradually adjusted to 40:60 (*v*/*v*) under gradient conditions and then maintained under isocratic elution. The detailed chromatographic conditions for separation and collection are presented in Table S2 of the Supplementary Materials.

#### 2.4.2. HPLC Confirmation of SFN-DX

The SFN-DX fraction collected via prep-LC was completely dried using a freeze-dryer (LP10; IlShinBioBase Co., Ltd., Dongducheon, Gyeonggi-do, Republic of Korea). To confirm the purity of the isolated compounds, HPLC-UV analysis was performed under the conditions listed in Table S1.

### 2.5. Structure Identification

#### 2.5.1. HS-SPME/GC-MS

Based on preliminary experiments, a solid-phase microextraction (SPME) fiber (DVB/CAR/PDMS) and a DB-WAX capillary column (30 m × 0.25 mm i.d., 0.25 μm film thickness; Agilent, Santa Clara, CA, USA) were selected to detect volatile sulfur compounds (VSCs) in SFN-DX. A 10 μL sample of freeze-dried SFN-DX in oily form was diluted with 200 μL of methanol and transferred to a headspace vial, followed by the addition of 3–4 mL of distilled water. The vial was equilibrated for 10 min at 45 °C in an autosampler with a temperature control function, and HS-SPME was performed. Volatile components in the headspace were adsorbed for 40 min using the DVB/CAR/PDMS fiber, desorbed at 250 °C for 10 min, and introduced into the gas chromatography injector port (split ratio: 10:1). Analysis was performed using a gas chromatography–mass spectrometry system (GC-MS; Agilent 8890A/5977B MSD Series, Agilent Technologies, Santa Clara, CA, USA) operated in full scan mode (*m*/*z* 35–550). The interface temperature was maintained at 230 °C, and ionization was performed using electron impact ionization (EI) at 70 eV. Separation of the SFN-DX volatiles was performed on a DB-WAX capillary column with helium as the carrier gas at a flow rate of 1.5 mL/min. The transfer-line temperature was set to 250 °C. VSCs in SFN-DX were identified by comparing their mass spectra to those in the NIST library as well as certified standards.

#### 2.5.2. Nuclear Magnetic Resonance (NMR) Analysis

The purified SFN-DX was dissolved in dichloromethane-d2 containing an internal standard and analyzed using both ^1^H and ^13^C NMR spectroscopy. Analysis was performed using a 600 MHz NMR spectrometer (JNM-ECZ600R, JEOL Ltd., Tokyo, Japan), and chemical shifts (δ) were reported in parts per million (ppm). To elucidate the detailed chemical structure of SFN-DX, additional two-dimensional NMR experiments, namely distortionless enhancement by polarization transfer (DEPT), heteronuclear single quantum coherence (HSQC), ^1^H–^1^H correlation spectroscopy (COSY), and heteronuclear multiple bond correlation (HMBC), were performed.

### 2.6. Storage Stability Evaluation

To assess the temperature-dependent stability of purified SFN-DX, samples were stored under four different temperatures, frozen (−18 °C), refrigerated (4 °C), ambient (20 °C), and elevated (40 °C), for either 1 week or 1 month. Stability was evaluated by monitoring the changes in compound purity using HPLC analysis, as described in Table S1. These results provide fundamental data for the optimization of storage conditions and long-term stability of SFN-DX.

## 3. Results and Discussion

### 3.1. HPLC Analysis

#### 3.1.1. Standard Curve of SFN

**Figure 1:**
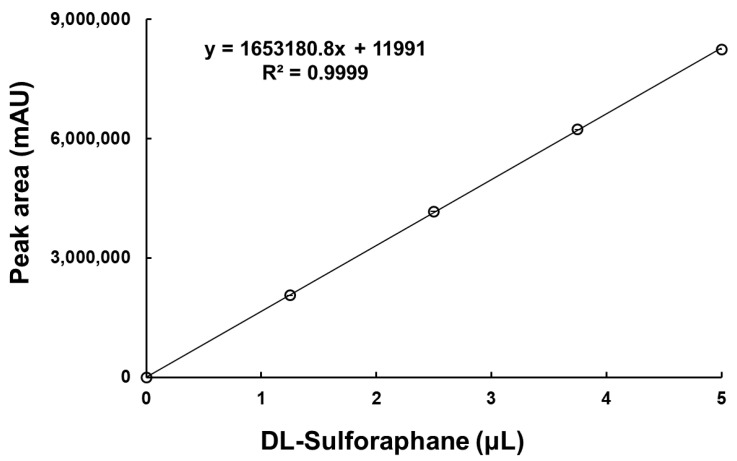
Calibration curve of DL-sulforaphane standard solution. Each data point represents the mean ± standard deviation (*n* = 3); error bars are present but are smaller than the symbol size and may not be visible.

To quantify the SFN-DX content in the components extracted from freeze-dried radishes, HPLC analysis was performed using an SFN standard. Linear regression analysis of SFN standard concentrations yielded a high correlation coefficient (R^2^ = 0.9999), demonstrating excellent linearity. The resulting regression equation was y = 1,653,180.8x + 11,991, and the standard calibration curve is shown in Figure 1. These results provide a reliable basis for the accurate quantification of SFN components in radishes. The SFN-DX content in freeze-dried radish was determined to be 13.262 mg/g.

#### 3.1.2. HPLC Analysis of SFN-DX Extracted from Radish

**Figure 2:**
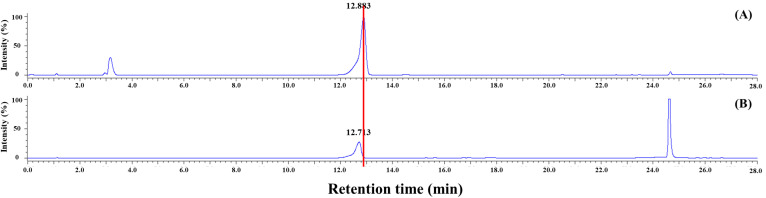
HPLC chromatograms of the SFN standard (**A**) and SFN-derived compound X (SFN-DX) extracted from freeze-dried radish (**B**), detected at 230 nm.

The HPLC chromatogram of SFN-DX extracted from radish prior to purification is shown in Figure 2. As shown in Figure 2A, the SFN standard (95% purity) exhibited a distinct peak at a retention time (RT) of 12.883 min. In contrast, SFN-DX extracted from radish (Figure 2B) exhibited two major peaks, including one at an RT of 12.713 min, closely matching the SFN standard. Radish contains sulfur-containing compounds, primarily from the glucosinolate family, which are enzymatically hydrolyzed by myrosinase into various secondary metabolites [3,26]. Representative metabolites include glucoraphasatin, glucoraphenin, glucobrassicin, and SFN [27,28], with their composition and abundance varying depending on the cultivar, processing method, and environmental factors such as altitude [29,30,31,32,33]. Although SFN-DX had a retention time similar to that of the SFN standard, its earlier elution suggests that it had a lower molecular weight. Therefore, SFN-DX was presumed to be a derivative or modified form of SFN, potentially with structural differences. To elucidate its chemical structure, SFN-DX was isolated and purified for structural characterization through GC-MS and NMR analyses 262 mg/g.

### 3.2. Separation, Purification, and Structural Elucidation

#### 3.2.1. Isocratic and Gradient Elution

**Figure 3:**
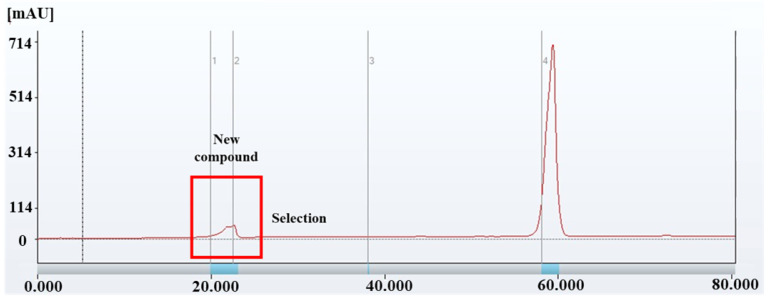
Prep-HPLC chromatogram of SFN-DX extracted from freeze-dried radish, detected at 230 nm. The chromatogram shows the isolation of SFN-DX with a retention time of approximately 20.0 min, followed by a major peak at approximately 60.0 min. The red box highlights the fraction selected for further purification and structural analysis.

**Figure 4:**
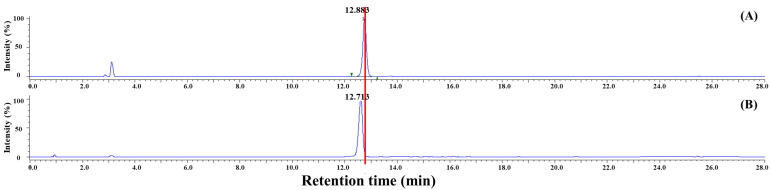
HPLC chromatograms of the components purified by Prep-HPLC at 230 nm absorbance: (**A**) purified SFN standard and (**B**) purified SFN-DX.

The chromatographic separation and fractionation results obtained through prep-LCs are shown in Figure 3. Two major peaks were observed (RT: 20 min and 60 min), consistent with the HPLC analysis. A single fractionation was performed targeting the peak at RT 20–23 min using a C18 column. Subsequent HPLC analysis of the collected fraction revealed peaks at RT 12.883 min (Figure 4A) and RT 12.713 min (Figure 4B), matching the SFN standard and the SFN-DX component extracted from radish, respectively. Purified SFN-DX was obtained as a brown, oil-like solid after freeze-drying. HPLC analysis confirmed 99% purity. These results demonstrate that high-purity SFN-DX was successfully isolated and purified from the radish extract, making it suitable for structural elucidation via GC-MS and NMR analysis.

#### 3.2.2. GC-MS

**Figure 5:**
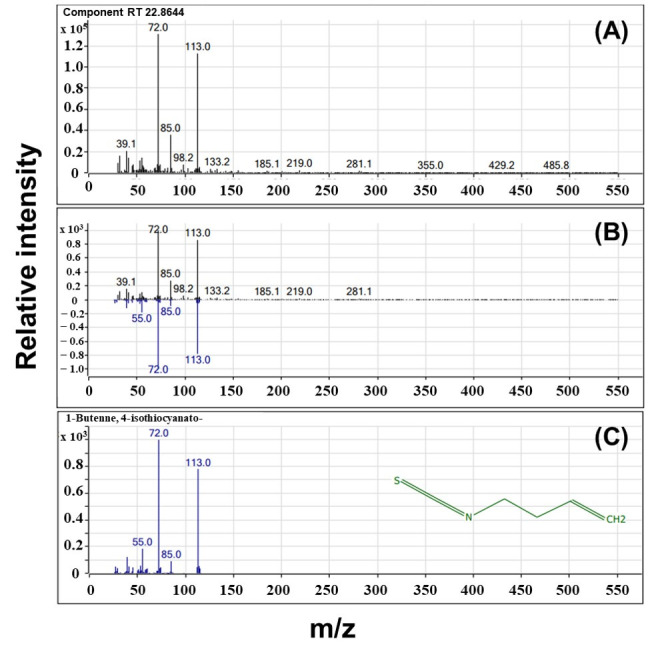
Gas chromatography–mass spectrometry (GC-MS) mass spectra of purified SFN-DX compared to 1-butene, 4-isothiocyanato-. (**A**) The top panel shows the mass spectrum of purified SFN-DX at a retention time of 22.8644 min, showing major peaks at *m*/*z* 39.1, 72.0, 85.0, 113.0, and 133.2. (**B**) The middle panel shows an overlay of the spectra of purified SFN-DX and the reference samples. (**C**) The bottom panel shows the reference spectrum for 1-butene, 4-isothiocyanato-.

The mass spectrometric profile of SFN-DX is shown in Figure 5. The EI-MS spectrum of SFN-DX exhibited prominent *m*/*z* peaks at 39.1, 72, 85, 113, 133.2, 185.1, 219.0, and 281.1. This profile closely resembled previously reported EI-MS spectra of SFN (*m*/*z*: 72, 39, 45, 55, 60, 64, 85, 114, 119, 160, 177 [M^+^, 1]) and 3-butenyl isothiocyanate (*m*/*z*: 72, 53, 55, 85, 113) in broccoli [34]. These compounds share a characteristic isothiocyanate (-N=C=S) functional group, indicating structural similarity with SFN and its analogs. Isothiocyanate compounds are characterized by high reactivity and their capacity to form diverse derivatives under diverse environmental conditions [35,36]. Therefore, SFN-DX is likely a novel isothiocyanate derivative of SFN.

#### 3.2.3. NMR

**Figure 6:**
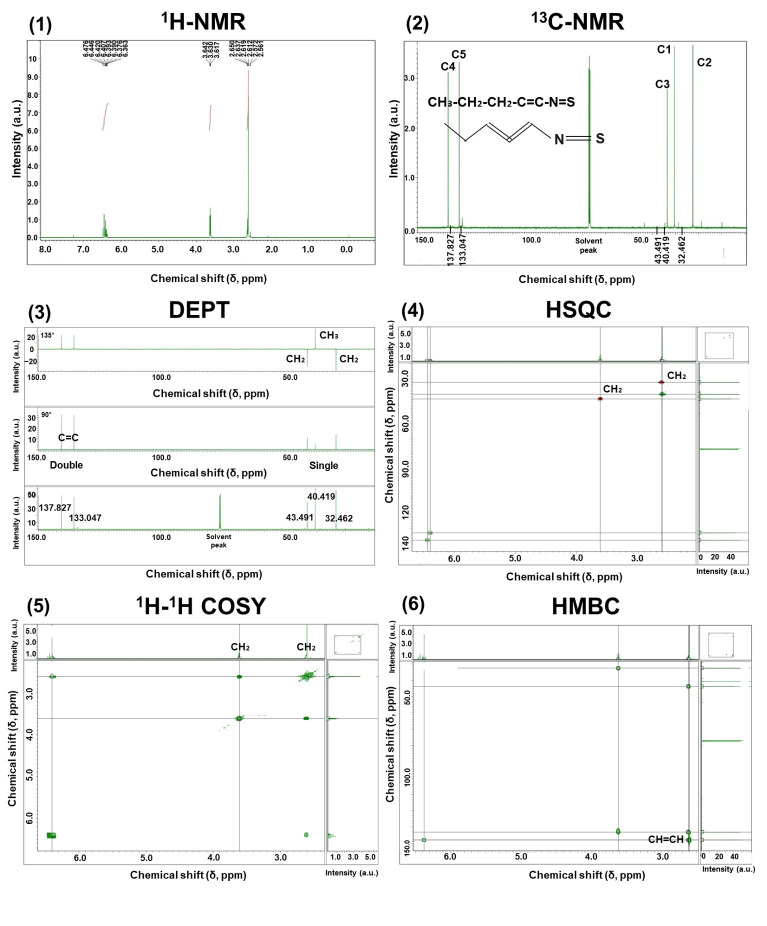
NMR spectra for the structural identification of purified SFN-DX: (1) ^1^H-NMR for proton analysis. (2) ^13^C-NMR for determining the carbon framework. (3) Distortionless enhancement by polarization transfer (DEPT) for distinguishing CH, CH_2_, and CH_3_ groups. (4) Heteronuclear single quantum coherence (HSQC) showing correlations between ^1^H and ^13^C. (5) ^1^H-^1^H correlation spectroscopy (COSY) for determining proton-proton interactions. (6) Heteronuclear multiple bond correlation (HMBC) for long-range heteronuclear correlations.

The final structure of purified SFN-DX was determined using ^1^H-NMR, ^13^C-NMR, DEPT, HSQC, ^1^H–^1^H COSY, and HMBC spectroscopy, as shown in Figure 6. Comprehensive analysis of the NMR data revealed that SFN-DX exhibited spectral features that were highly consistent with those of SFN, as reported on sulforaphane extracted from broccoli by Kore et al. [37] and Zhang et al. [38], strongly suggesting that SFN-DX is a sulforaphane-derived compound. SFN belongs to the isothiocyanate family, which includes highly reactive compounds that can undergo structural transformations in response to environmental factors, such as oxygen and light. In this study, the isolated compound was determined to possess the structural formula CH_3_–CH_2_–CH_2_–CH=CH–N=S, further validating its classification as an SFN derivative. This structural characterization is consistent with previous findings on isothiocyanate extracts from cauliflower, radish, and radish seeds [6,17].

### 3.3. Stability of Purified SFN-DX at Different Temperatures and Storage Periods

**Figure 7:**
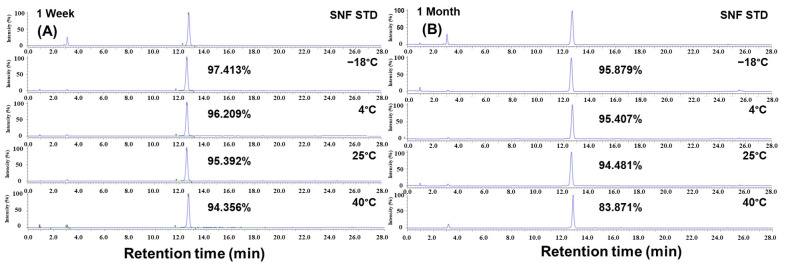
Storage stability of purified SFN-DX at different temperatures and durations. HPLC chromatograms of purified SFN-DX after 1 week (**A**) and 1 month (**B**) of storage under various temperatures, detected at 230 nm. The corresponding purity values of SFN-DX are indicated on each chromatogram.

The stability of purified SFN-DX at various storage temperatures and durations was evaluated, and the results are shown in Figure 7. Samples were stored at −18 °C, 4 °C, 20 °C, and 40 °C, and analyzed after 1 week and 1 month using the HPLC method outlined in Table S1. The purity of SFN-DX decreased significantly as the storage temperature increased. After 1 week, the highest purity was observed at −18 °C (97.413%) and progressively declined at 4 °C (96.209%), 20 °C (95.392%), and 40 °C (94.356%). Following 1 month of storage, the purity decreased further to 95.879% at −18 °C, 95.407% at 4 °C, and 94.481% at 20 °C, with a significant drop to 83.871% at 40 °C. Overall, both higher storage temperatures and longer durations led to a greater reduction in the purity of SFN-DX, with the most pronounced losses observed under elevated temperature conditions. These results suggest that SFN-DX is thermally unstable and susceptible to degradation or chemical transformation at elevated temperatures, likely owing to the inherent reactivity of the isothiocyanate functional group. Similar findings were reported by Oh. [39], who observed a greater loss of bioactive compounds with increasing extraction temperatures. While it is generally recognized that low-temperature storage enhances stability, the present study provides quantitative evidence of purity loss across multiple storage temperatures and over time, thereby underscoring the necessity of maintaining controlled storage conditions for sulforaphane-derived compounds. Collectively, these results indicate that storage at −18 °C is optimal for preserving the structural and chemical integrity of SFN-DX, providing a critical guideline for preserving physiological activity as a functional ingredient.

## 4. Conclusions

In this study, SFN-DX, a novel dietary sulfur compound, was successfully extracted and purified from freeze-dried radish. Comprehensive analyses leveraging HPLC, prep-LC, GC-MS, and NMR confirmed the identity and structure of SFN-DX. The compound exhibited an RT comparable to that of the SFN standard in HPLC analysis, and its structure, characterized by the presence of an isothiocyanate moiety, was further elucidated through GC-MS and NMR analyses. Specifically, NMR data confirmed an alkyl-modified structure with the formula CH_3_–CH_2_–CH_2_–CH=CH–N=S, confirming SFN-DX as a sulforaphane-derived compound. Stability analysis revealed that SFN-DX is temperature-sensitive, exhibiting marked degradation at higher storage temperatures and over prolonged durations. Among the evaluated conditions, storage at −18 °C yielded the highest stability, suggesting that low-temperature preservation for maintaining compound integrity. Although the bioactive potential of SFN-DX is suggested based on its structural similarity to sulforaphane, direct biological activity tests were not conducted in this study. Future research will therefore focus on experimental validation of antioxidant and biological activities through in vitro, in vivo, or in silico approaches, as well as exploring potential applications of SFN-DX in functional and health-promoting foods. Therefore, this study provides scientific evidence supporting the extraction, structural identification, and optimal storage conditions of this novel sulforaphane-derived compound, while laying the groundwork for its further evaluation and utilization in food science and nutrition.

## Data Availability

The original contributions presented in the study are included in the article, further inquiries can be directed to the corresponding author.

## Supplementary Materials

The following supporting information can be downloaded at: https://www.mdpi.com/article/10.3390/foods14183254/s1. Table S1. HPLC operating conditions for the identification of the SFN standard and SFN-DX components extracted from freeze-dried radish. Table S2. Prep-HPLC operating conditions for the separation and purification of SFN-DX components extracted from freeze-dried radish.

## Abbreviations

The following abbreviations are used in this manuscript:

SFN	sulforaphane
SFN-DX	SFN-derived compound X
RT	retention time
HPLC	high-performance liquid chromatography
DW	distilled water
ACN	acetonitrile
HS-SPME	headspace solid phase microextraction
GC-MS	gas chromatography–mass spectrometry system
NMR	nuclear magnetic resonance
DEPT	distortionless enhancement by polarization transfer
HSQC	heteronuclear single quantum coherence
COSY	^1^H–^1^H correlation spectroscopy
HMBC	heteronuclear multiple bond correlation
EI-MS	electron ionization–mass spectrometry
