# Supplementary material for: Structural Elucidation and Storage Stability of Novel Dietary Sulfur Compounds from Radish
Source: Foods. 2025 Sep 19;14(18):3254. doi: 10.3390/foods14183254 (PMC12469551; doi:10.3390/foods14183254)
Supplement: Supplementary file 1 [file foods-14-03254-s001.zip › foods-3827187-supplementary.pdf]

Supplementary Materials

Table S1. HPLC operating conditions for the identification of the SFN standard and SFN-DX components extracted from freeze-dried radish.

HPLC-UV	Jasco LC-4000		
**Column**	C18 (250 mm × 4.6 mm × 5 μm, YMC-Pack ODS-AQ)		
**Mobile phase**	A: Acetonitrile (ACN) B: DW (Distilled water)		
**Detector**	UV-vis detector (230 nm)		
**Run time**	28 min		
**Flow rate**	1 mL/min		
**Injection volume**	10 μL		
**Column temperature**	30 °C		
**Method conditional (gradient)**	**Time**	**Mobile phase A (%)**	**Mobile phase B (%)**
	00.00	19	81
	05.00	19	81
	20.00	60	40
	20.01	100	0
	25.00	100	0
	25.01	19	81

Table S2. Prep-HPLC operating conditions for the separation and purification of SFN-DX components extracted from freeze-dried radish.

HPLC-UV	LC-Forte/R-II, YMC		
**Column**	C18 (20 mm × 500 mm × 15 μm, JAIGEL-ODS-BP-L, JAIGEL)		
**Mobile phase**	A: Acetonitrile (ACN) B: DW (Distilled water)		
**Detector**	UV-vis detector (230 nm)		
**Run time**	100 min		
**Flow rate**	20 mL/min		
**Injection volume**	10 mL		
**Column temperature**	30 °C		
**Method conditional (gradient)**	**Time**	**Mobile phase A (%)**	**Mobile phase B (%)**
	00.00	19	81
	44.00	19	81
	80.00	60	40
	80.02	60	40
	96.00	100	0
	96.01	100	0
